# Effects of long-term adapted physical training on functional capacity and quality of life in older adults with Parkinson's disease

**DOI:** 10.3934/Neuroscience.2024028

**Published:** 2024-11-27

**Authors:** Oussama Gaied Chortane, Elmoetez Magtouf, Wael Maktouf, Sabri Gaied Chortane

**Affiliations:** 1 Research Unit (UR17JS01) Sports Performance, Health & Society, Higher Institute of Sport and Physical Education of Ksar-Said, Universite de La Manouba, Tunis 2010, Tunisia; 2 Bioengineering, Tissues and Neuroplasticity, UR 7377, Faculty of Health, University of Paris-Est Créteil, 8 rue du Général Sarrail, 94010 Créteil, France

**Keywords:** Parkinson disease, older adults, physical activity, quality of life, motor function

## Abstract

**Background:**

Parkinson's disease (PD) remains incurable and its prevalence is increasing as the population ages. Although physical activity is considered a therapeutic treatment to slow the progression of the disease, it is considered to be an effective non-pharmacological adjuvant to medication to improve the symptom management.

**Methods:**

The training program was offered for all the participants (N = 50) in three non-consecutive sessions per week for 60 minutes and a total duration of 12 to 16 months. Each session is composed of warming up, adapted boxing training exercises, muscle building and resistance exercises, and returning to calm. For the measurement of physical capacities, the following tests were administered: the Fullerton Advanced Balance Scale (FAB), Timed Up and Go (TUG), and the 30-second chair lift test (TLC30). With regard to quality of life, the Parkinson's Disease Questionnaire of 39 questions (PDQ-39) was used. The participants (age range from 60 to 80 years) were divided following the results of the Parkinson disease severity (Questionnaire Hoehn and Yahr; H&Y) into two groups (H&Y 1–2: mild to moderate symptoms; H&Y 3–4: moderate to severe symptoms).

**Objective:**

The aim of this research was to assess the long-term effects (12 to 16 months) of a community-wide adapted physical program on the physical capacity and quality of life of people with Parkinson disease.

**Conclusion:**

In view of the results, adapted physical training appears to be beneficial for physical capacity and life quality and considered to be an important approch for maintaining the physical and mental capacities and slowing down the proression of neurodegenrative disease.

## 1. Introduction

Neurodegenerative diseases are likely to be a scourge for our society in the course of the next century. Parkinson disease (PD) is the second most common neurodegenerative disease after Alzheimer disease [1], and it is characterized by the gradual worsening of various motor and cognitive symptoms [2]. The latter are often subtle at the onset and can occur many years prior to a diagnosis in different brain regions, which makes the sympthomalogy of the disease inivitable. Due to the progression of the pathology, physical parameters will be infected, such as walking and balance, and is accompanied by cogntive deficits, which will negatively impact the patient's quality of life [3],[4]. Therefore, pharmacological treatments are aimed at alleviating symptoms and improving functional capacities. They are mainly focused on the dopaminergic deficit of the black substance (i.e., the main anatomical characteristic of the pathology) [5]. For this purpose, levodopa has remained the leading treatment used, especially in the early stages of the disease. However, long-term use may lead to an intolerance, thus requiring increased doses, and sometimes causes motor fluctuations called dyskinesies, thus increasing the risk of falling and affecting quality of life (QoL) [6].

Exercise has long been postulated as a theraputique intervention that can modify the short- and long-term clinical courses of patients with PD [7],[8]. Conversely, people with PD display low levels of physical activity and a prolonged sedentary behavior [9], which may negatively impact the clinical course of the disease [10]. Consequently, identifying and developing specific procedures to increase PA levels in this population is critical. Because exercise is a multidimensional activity with combined effects on both the physical and mental wellbeing, it would be more reasonable to assess the impact of exercise by measuring QoL changes. Indeed, several researchers have shown that exercise plays an important role as a supplement to drug therapy in relieving suffering and improving the QoL [11]. QoL outcomes are useful for attaining evidence of meaningful benefit, thus enhancing the significance of variables of interest and allowing for more holistic decision-making [12]. The link between QoL and physical activity has been increasingly consolidated in the literature [13], including in PD, whereby many studies have assessed various non-pharmacological treatments, including physical rehabilitation programs [14], Tai Chi Quan [15], and aquatic physiotherapy [16], which demonstrated an improvement in a patient's QoL and well being with the use of specific instruments to assess the QoL in this population [17].

In practice, research with people with PD has been on the rise in the last 10 years. While adapted physical programs based on boxing and resistance exercices are emerging, few studies have documented its effectiveness. Furthermore, many of the studies conducted with people with PD did not necessarily take medication changes and the severity of the disease into account. Eventually, few interventions have been carried out over a period of more than a year, while PD progresses slowly. In light of these findings, the overall objective in the present study is to assess the long-term effects of adapted physical training (12 to 16 months) on the physical capacities and QoL in older adults with PD.

## 2. Materials and methods

### 2.1. Participants

The present study was aimed at people with PD who lived in the Monastir region, were enrolled in the adapted physical program, were aged between 60 and 80 years, and had no severe cognitive decline that limited their ability to make an informed decision. At first, fifty individuals participated; of these, 40 enrolled 12 to 16 months before the study was completed. A total of eight people were either inactive or had left the program before reassessment. Three individuals were excluded because of a different diagnosis of idiopathic PD (Lewy body dementia) (n = 2) and essential tremors (n = 1), while one participant who was involved as a co-researcher in this study was also excluded due to his status on the team. Finally, one participant could not be re-evaluated in time because of the sanitary confinement caused by COVID-19, and another refused to sign the consent paperwork.

Of the 28 potential participants, 26 provided their consent to participate in the study. The subjects were informed about the experimental protocol and signed a written consent form according to the standards and guidelines of the Protection Committee of the University of Monastir (Tunisia).

### 2.2. Ethical Statement

All participants gave their informed consent for inclusion before they participated in the study. The research was conducted according to the guidelines of the Declaration of Helsinki and approved by the ethical advisory committee of the Research Unit (UR17JS01) Sports Performance, Health and Society, Higher Institute of Sport and Physical Education of Ksar Saîd, University of Manouba, Tunis, 2010, Tunisia. Additionally, all procedures that were performed with human participants were in accordance with the ethical standards. The study protocol was accepted by the Ethical Commission of Manouba University in April 2023 (Ref: HE24FEB2006-B223822).

### 2.3. Physical Training Program

The selected physical program is composed of 3 sessions per week. Hoehn & Yahr (H&Y) 1–2 participants had the option of inviting a close caregiver (called cornerman) to either assist them or participate alongside them, while H&Y 3–4 participants were almost always accompanied for safety reasons. Furthermore, the training structure used an appropriate approach to the participants according to their health condition: the exercises were adjusted according to physical limitations (e.g., total hip prosthesis) or signs of severity of the disease (e.g., risk of loss of balance and fall), which included wearing a belt on the waist, praticing the exercise, and sitting in a wheelchair. At the beginning of each course, the participants put elastic bandages around their hands and wrists to support their joints.

Each session was composed as follows: 10 minutes warm-up on the tredmill with a 3 to 5 klm/h speed, which indicated a normal to moderate intensity (50–65 bpm/min); twenty minutes of adapted boxing exercises on a bag with right and left hands in two situations—static and dynamic—separated by interval training; twenty minutes of balance and gait training using a Bosu Ball, a medicine ball (1 kg), and elastic bands (10 and 15 kg); and a return to calm (5–10 minutes) with a static stretching of the muscles at various joints (elbows, knees, shoulders, neck, wrists, ankles, and hips) and various breathing techniques. We encouraged the participants to adopt a healthy and active lifestyle, thereby allowing them to attend classes once, twice, or three times a week without any restrictions on their outdoor activities. At each meeting, they need to sign a presence register to assess their attendance.

### 2.4. Inclusion and Exclusion cretiria

Eligible individuals for this study included people with PD who participated in the adapted physical program for at least one year, had a reassessment, and consented to their data being extracted from their medical file. However, individuals with secondary or atypical parkinsonism were excluded (Lewy body dementia and essential tremors). Finally, those who were unable to complete the reassessment due to health problems were also excluded.

### 2.5. Evaluation of Physical Capacity

Postural control and balance were evaluated with the Fullerton Advanced Balance Scale (FAB) test to assess the risk of falling [18]. Ten tasks needed to be completed and were rated by the appraiser on a 5-point scale (0 to 4) with specific achievement criteria according to the task. A maximum score of 40 points indicated a high performance, and this test is one of the most standardized test for people with PD, with an excellent interjudge and test-retest fidelity (CCI [3],[1] ≥ 0.95) [19],[20].

Mobility was evaluated with the Timed Up and Go (TUG) test, which is norally validated in people with PD [21]. The participant started the test on a standard 48.5 cm high chair with armrests, in which the participant's back touched the back of the chair and their feet touched the floor. At the evaluator's signal, the participant rose from the chair, which started from a starting position with their hands on the armrests, bypassed a cone placed in front of the seat at a distance of three meters, and returned to reassure themself on the chair as quickly as possible in a safe manner. The TUG test-retest is a beneficial tool for people PD (r = 0.73–0.99; CCI [3.1] = 0.87–0.99) [21]. The Minimal Detectable Change (MDC) for this population varied widely between 2 and 11 seconds. A previous study combined the TUG with a dynamic walking index and established a 3.5-second MDC; however, it did not include participants with an initial score greater than 20 seconds [22].

The endurance and strength of the lower limbs were measured using the 30-second chair lift test (TLC30), which is a valid test for people with PD [23]. The participant started the test sitting on a chair at a standard height of 48.5 cm without arm support, with their arms crossed on the chest and their hands on the shoulders. Then, the participant rose and elevated the chair completely for 30 seconds, and tried to perform as many repetitions as possible. If a replay was already started in the 30th second, it was compiled. The 30-second chair lift is a validated test to detect changes over time in people with PD and has moderate to excellent test-retest and interjudge reliability (CCI [2.2] = 0.94) [24],[25].

Finally, the strength of the upper limbs was evaluated with a tension strength test (Digital Dynamometer EH101-17; Camry, Seattle, WA, USA) during the reassessment so that the data could be used in further research and the sample could be compared to those in the literature. The participant took the device, adjusted it to the size of their hand so that it had a 90-degree angle to the proximal phalanges, and fixed it upright, with foot-wide shoulders and their arms along the body. Two hand trials were conducted, and the best score for each hand was compiled.

### 2.6. Health-related quality of life

QoL was evaluated with the 39-question Parkinson's Disease Questionnaire (PDQ-39). This is a valid tool specially designed to measure the effects of PD on the QoL [26]. This is a questionnaire comprised of 39 questions divided into eight categories: mobility, daily life activity, emotions and well-being, stigma, social support, cognition, communication, and physical pain. The respondent chose a response based on the last 30 days, which qualified their perception for each statement and results in a score between 0 and 4. A total score is used, and a maximum of 100 indicated the lowest possible QoL.

### 2.7. Evaluation of Parkinson disease Severity

This classification is based on the H&Y scale, which makes it possible to classify participants according to their motor symptoms. For example, a score of 1 shows only a unilateral injury, usually with or without a functional impairment, whereas a rating of 4 is a severely disabling injection, though the individual is still able to walk or stand without help. The classification of the participants in the H&Y 1–2 and H&Y 3–4 groups was performed by a clinical nurse and associate professor at the University of Monastir. Furthermore, the age and time factors of the participants are particularly important variables for the initial diagnosis and can generally reflect a more advanced degree of severity [27].

### 2.8. Program attendance

Program attendance was assessed through the participant's presence during the physical training sessions and was recorded in computer databases. The attendance percentage reflected the number of courses a person attended based on the total number of classes offered individually (taking public holidays into account).

## 3. Data analysis

The sample size of our population was small, and the normality of the data was checked with the Shapiro-Wilk test and the visual histogram analysis [28]. Statistical analyses were unable to demonstrate a normal distribution for all data, and the measurements are presented in medians and interquartile extents. In addition, non-parametric tests were used to analyze the data. Wilcoxon-signed rows were used to meet the principal objective and to assess the effect of the program on the variables under study (physical capacity and quality of life) for all participants, as well as in each group separately (H&Y 1–2 and H&Y 3–4), in order to consider the severity of the PD. To assess whether the severity level influenced the changes induced by the physical program, a comparison of delta changes (revaluation value and initial evaluation value) of the groups was performed with the Mann-Whitney U test. The Chi-Square test was used to compare the frequencies of qualitative data between groups. The significance threshold was set to p ≤ 0.05, and the analysis was performed using the SPSS software, version 25.0 (IBM).

## 4. Results

### 4.1. Characteristics of participants

The study sample included 26 participants with a diagnosis of PD, who lived in Monastir region, and mostly suffered from chronic comorbidities. No serious events occurred during the training; additionally, 17 of the 26 participants (65.4%) had joint pain and movement limitations. The program attendance rate was 58% for all 26 participants. The engagement was slightly higher in H&Y groups 3–4, with 69% versus 57% for H&Y groups 1–2. Table 1 presents the characteristics of the participants, their attendance at the physical program, their resilience to reassessment, and their comorbidities. There is a significant difference between the two groups in terms of age and comorbidities.

**Table 1.** Characteristics of participants.

Groups	Total Population (N=26)	H&Y 1-2 (N=20)	H&Y 3-4 (N=6)	Group Comparison (p)
Age (years)	69 (47-82)	67 (47-82)	76 (64-77)	.011
65 years and under (n (%))	8 (31)	7 (35)	1 (17)	.393
65 ans and over (n (%))	18 (69)	13 (65)	5 (83)	.392
Time since diagnosis (Year)	4,9 (1,3 – 16,7)	4,8 (1,3 – 16,7)	6 (3 – 16,1)	.301
Days between evaluations	431 (406 – 467)	427 (385 – 478)	451 (423 – 501)	.144
Men (n (%))	16 (62)	13 (65)	3 (50)	.516
Levodopa (mg/day)	500 (100 – 950)	475 (100 – 900)	725 (450 – 950)	.058
Number of meetings attended	61 (47 – 76)	60 (37 – 75)	69 (57 – 86)	.201
Assiduity (%)	58 (19 – 85)	57 (19 – 85)	69 (44 – 78)	.377
Strength of strain (kg)	63.1 (48.2 – 75.2)	63.8 (49.8 – 63,8)	48.7 (45.8 – 70.6)	.224
Hypotension (n (%))	7 (27)	5 (25)	2 (33)	.686
Type 2 diabete (n (%))	3 (12)	0 (0)	3 (50)	.001
Thyroid gland problem (n (%))	6 (23)	4 (20)	2 (33)	.497
Cardiovascular history (n (%))	4 (15)	2 (10)	2 (33)	.165
Cancer (n (%))	3 (12)	1 (5)	2 (33)	.567
Physical limitations (n (%))	17 (65)	14 (70)	3 (50)	.366

### 4.2. Physical capacity

#### 4.2.1. Mobility

The time required to complete the TUG for all participants decreased from 7.3 to 6.0 seconds (p = 0.0001). In each group, a significant reduction was observed (H&Y 1–2: 6.6 to 5.1 seconds, p = 0.0001; H&Y 3–4: 10.1 to 7.3 seconds, p = 0.028). For all groups, 24 participants (92.3%) improved their scores, while 2 participants (7.7%) took more time to complete the task, with 0.30 and 0.78 seconds within a possible margin of error, respectively (Minimal Detectable Change between 2 and 11 seconds).

#### 4.2.2. Strength of lower limbs

The maximum number of performed repititions in TLC30 for all participants improved significantly from 12.0 to 14.5 (p = 0.001). In the H&Y 1–2 group, the number of repetitions increased from 12.0 to 15.0 (p = 0.003) and from 10.5 to 13.0 in the H&Y 3–4 group, but was not significant (p = 0,10). The percentage of improvement was 80.8%.

#### 4.2.3. Static and Dynamic Balance

The FAB results remained unchanged in all groups (p = 0.80), as well as in each group separately (H&Y 1–2: p = 0,59; H&Y 3–4: p = 0.92). The physical performance results are summarized in Table 2.

**Table 2.** Physical capacity before and after the physical intervention.

Group	Total Population (N = 26)	H&Y 1-2 (n = 20)	H&Y 3-4 (n = 6)	Group Comparison (p)
TUG (sec)				
Before	7.3 [6.2– 8.6]	6.6 [5.8 – 8.0]	10.1 [8.0 – 15.9]	.006
After	6.0 [4.9 – 6.8] *	5.1 [4.7 – 6.4] *	7.3 [8.0 – 15.9] *	.003
∆	- 1.8 [-2.3 – -0.7]	-1.6 [-2.1 – -0.7]	-2.4 [-5.9 – -1.8]	.024
TLC30 (rep)				
Before	12.0 [10.8 – 14.3]	12.0 [11.3 – 15.0]	10.5 [8.8 – 11.5]	.023
After	14.5 [12.8 – 17.3] *	15.0 [14.0 – 17.8] *	13.0 [9.8 – 15.0]	.098
∆	3.0 [0.0 – 4.0]	3.0 [0.3 – 4.0]	2.5 [-0.3 – 4.8]	.806
FAB (score)				
Before	34.5 [29.0 – 38.0]	36.5 [31.5 – 38.0]	24.0 [18.5 – 31.0]	.005
After	33.5 [29.5 – 38.0]	36.5 [30.5 – 38.0]	26.5 [16.8 – 34.5]	.026
∆	0.0 [-3.3 – 3.0]	0 [-2.8 – 1.8]	+1 [-6.8 – 7.3]	.783

* = p ≤ 0,05: Significant difference from the initial values (Wilcoxon signed range test). Comparison between groups performed with Mann-Whitney U tests. Values are presented in median [interquartile length].

### 4.3. Quality of life

The overall PDQ-39 score significantly increased for both groups, thus suggesting a decrease in the QoL. In Table 3, a significant alteration is noted by an increase in the total score (p = 0.05), with changes in the dimensions of stigma (p = 0.006) and communication (P = 0.016). The other sub-scales showed no difference. Furthermore, only the participants in H&Y group 1–2 showed a significant increase in the total score (p = 0.02), as well as changes in the same dimensions of stigma (p = 0.009) and communication (p = 0.0013), in addition to the field of cognition (p = 2.09). Since there were three incomplete questionnaires during the initial evaluation, it is important to clarify that the comparison results before and after are based on the analysis of the results of 23/26 participants.

**Table 3.** Evolution of quality of life as a result of intervention.

Variables		Total Population (N = 26)	H&Y 1-2 (N = 20)	H&Y 3-4 (N = 6)	Group Comparison (p)
TUG	Before	12.3 [8.9 – 16.2]	10.2 [8.7 – 14.4]	26.1 [17.3 – 36.2]	.006
	After	16.1 [12.1 – 24.4] *	16.1 [11.8 – 23.3] *	17.3 [11.9 – 32.6]	.503
Mobility	Before	5.0 [0.0 – 20.0]	2.5 [0.0 – 10.0]	46.3 [10.6 – 66.9]	.108
	After	10.0 [0.9 – 17.5]	7.5 [0.0 – 12.5]	18.8 [15.0 – 31.3]	.009
AVQ	Before	20.8 [12.5 – 25.0]	20.8 [8.3 – 20.8]	37.5 [21.9 – 43.7]	.043
	After	12.5 [4.2 – 21.9]	12.5 [4.2 – 20.8]	12.5 [3.1 – 43.8]	.806
Emotional well-being	Before	16.7 [12.5 – 25.0]	16.7 [8.3 – 20.8]	20.8 [13.5 – 37.5]	.324
	After	16.7 [8.3 – 29.2]	16.7 [8.3 – 25.5]	29.2 [8.3 – 38.5]	.199
Stigma	Before	0.0 [0.0 – 12.5]	0.0 [0.0 – 6.25]	6.3 [0.0 – 21.9]	.397
	After	12.5 [0.0 – 18.8] *	12.5 [0.0 – 18.75] *	3.1 [0.0 – 23.4]	.592
Social support	Before	0.0 [0.0 – 0.0]	0.0 [0.0 – 0.0]	0.0 [0.0 – 12.5]	.246
	After	0.0 [0.0 – 8.3]	0.0 [0.0 – 3.1]	8.3 [0.0 – 27.1]	.037
Cognition	Before	12.5 [6.3 – 31.3]	12.5 [6.3 – 18.8]	34.4 [31.3 – 46.9]	.003
	After	18.8 [6.3 – 39.8]	18.8 [6.3 – 51.6] *	15.6 [6.3 – 28.1]	.480
Communication	Before	0.0 [0.0 – 16.7]	0.0 [0.0 – 8.3]	16.7 [0.0 – 39.6]	.215
	After	8.3 [8.3 – 33.3] *	8.3 [8.3 – 31.2] *	16.7 [6.2 – 41.7]	.639
Physical discomfort	Before	25.0 [16.7 – 41.7]	25.0 [16.7 – 41.7]	37.5 [25.0 – 65.6]	.131
	After	37.5 [16.7 – 50.0]	33.3 [16.7 – 50.0]	41.7 [20.8 – 50.0]	.902

= p ≤ 0,05: Significant difference from the initial values (Wilcoxon signed range test). Comparison between groups made with Mann-Whitney U tests. Values are presented in median [interquartile length].

### 4.4. Self-reported perception of symptoms

**Figure 1.**
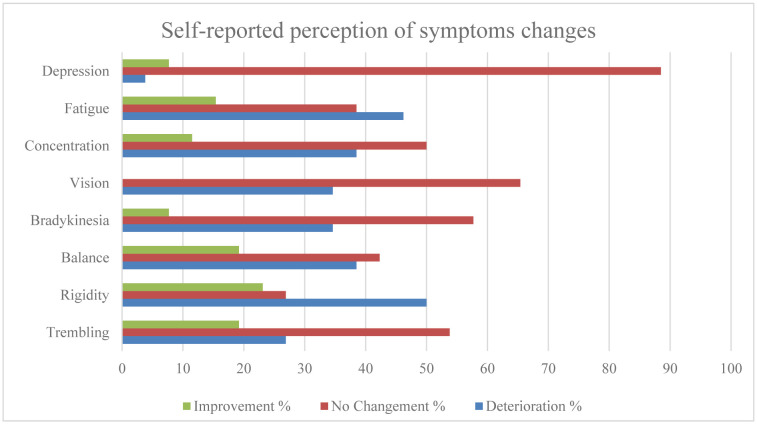
Perception of sympthoms changes.

With regard to the perception of the symptoms of the participants, Figure 1 presents detailed results and differences between the groups. In total, 34.1% perceived a deterioration, 52.9% peceived no change, and 13.0% perceived an improvement.

## 5. Discussion

The principal objective of this study was to assess the long-term effects of a physical training program on the functional capacity and QoL of people with PD. The results of this study should be interpreted with caution, as they highlight the benefits of long-term participation in a physical training program on motor function and life quality in older participants with mild and moderate level of PD.

### 5.1. Motor Symptom Benefit

Older peoples with PD invariably experience functional declines in a number of motor domains including posture, balance, gait, and transfers. After 12 to 16 months of participation in the program, the effects appear to be primarily beneficial for mobility, including a reduction in the time required to complete the TUG and an increase in the endurance of the lower limbs (increased number of rehearsals in the TLC30). While 24/26 participants improved their TUG scores, only 11/26 (42.3%) exceeded the minimum 2-second MDC. Regarding the endurance of the lower limbs, 15/26 (57.7%) exceeded the Minimal Detectable Change (TLC30: 3 rehearsals). However, this improvement did not result in changes in their static and dynamic balances (FAB). In the context of the Parkinson disease, this absence of change after 12 to 16 months could be regarded as an important maintenance: 46.2% of the participants improved their score within the values and 7/26 (26.9%) exceeded the MDC (2.25 points).

These findings are consistent with the literature that assessed the effects of a short-term training program and therefore support the role of a physical program on the motor capacity, which is an important component of functional capacity despite the evolution of PD. However, the absence of a significant balance benefit is surprising, as it differs from the study of Combs et al. [17], which displayed an improvement in balance, as measured with the Berg Balance Scale after a 12-week intervention (24 to 36 sessions). In comparison, our 12- to 16-month study resulted in a median participation of 61 sessions. Apart from the progression of the disease, the difference in the balance results could be explained, in part, by the volume of training. Indeed, if we compare the average of 30 sessions for 12 weeks (rate of 2.5/week) versus 61 sessions for 56 weeks (ratio of 1.09/week), we can understand that the participants in our study did not complete the same training load, and therefore did not meet the recommendations of Gallo et al. 2011 [29]. This difference is probably explained by multiple reasons, because the minimum number of sessions was already one of the exclusion criteria for the study.

In the study of Mehrholz et al. [30], the results showed that treadmill training improved balance, gait, and lead to important improvments of the walking parameters. Herman's research review concluded that treadmill training should play an essential role in improving gait and mobility in the care of individuals with PD [31]. However, resistance training (RT) is a relatively new technique for older people with neurodegenerative diseases. Saltychev et al. [32] emphasized the importance of resistance and strength training in people with PD. Furthermore, few studies have shown a positive effect of RT using medicine ball and elastic band exercises on the muscle strength and mobility of patients with PD [33]. A multicomponent physical program based on aerobic and strength exercise interventions was shown to be an effective method to delay the progression of PD. Thus, to make definite clinical recommendations on the possible use of resistance training for patients with PD, further studies should concentrate on larger sample sizes with sufficient follow-up periods.

Among the various AP interventions offered to people with PD, the contactless adapted boxing emerged as a non-traditional sensor motor training for functional capacity. This type of training is of interest to those affected, as it could help to improve several components of physical capacity affected by PD [34],[35]. Only a few studies have assesed the effects of adapted boxing treaining and resistance exercices on the motor function of aged people with PD [36]. Physical exercise represents a complementary treatment option, yet previous studies have failed to demonstrate its uniform benefit on mobility [37],[38].

The present reseach has a much greater ecological validity since the intervention was offered in the community environment without a control for assiduity. The objective was to improve the motor function, and the presented results here reflect the reality of a community program, which leaves a free choice for participation to improve their well-being. However, if a certain amount of training is needed to balance the benefits and reduce the significant risk of falling in this population [39], it would be important to advise the participants and their cornermen who enroll in this type of program. However, further research is needed to determine whether an optimal volume of suitable boxing training exists for this population. Scientific research has mainly focused on identifying PA barriers among patients with PD; however, there is a paucity of research on other relevant factors related to the PA behavior. For instance, there is a lack of knowledge regarding the pre-diagnosis PA behavior of people with PD, which may be useful in understanding their physical activity engagement [40]. Similarly, additional studies are necessary to investigate the relationship betwen PA levels and factors such as the QoL or the impairment level [41].

### 5.2. Cognitive Symptom Benefit

Concerning the QoL, a slight decrease (PDQ-39) was observed at the time of reassessment. This change seems to be mainly linked to the decline in stigma, communication, and cognition. In the absence of a monitoring group, it remains difficult to know the extent of this decline. It is possible that the reason of this decrease could have been much larger. Evidence from previous studies regarding the influence of exercise on QoL considerably varies. Moreover, a study that followed 728 people with Parkinson disease for 6 months displayed a decline in all dimensions of the QoL [42]. When we analyzed the changes in the present study before and after the physical intervention, it seems that the physical program did not slow down the decline in the QoL that occurs with PD. Indeed, the decline observed for the cognitive dimension was greater for the H&Y group 1–2, which can be explained by several factors. This may be due to the natural progression of the disease; however, other possibilities exist that can play a role in either increasing or decreasing the QoL, such as a different diagnosis of PD among the participants in the H&Y group 1–2 and other factors such as the environment (everyday tasks).

Furthermore, it is also necessary to mention some factors releated to the decline of certain activities under the selected physical program. In fact, the warm-up often contained an activity that required the participants to speak in front of the group; two aspects, namely the ability to speak and the pronounciation, may decrease with the disease [43]. Although the adapted physical program was aimed at breaking isolation and promoting socialization, the decline observed in the various dimensions could be explained by the discomfort of some participants in exposing their symptoms to the rest of the group. Moreover, studies report that people with PD can sometimes experience a sense of shame about their language difficulties and the visibility of their condition [44]; however, it remains desirable to offer opportunities to verbally expess themselves and to promote communication with peers in order to break the isolation in this population, since patients with Parkinson's or Alzheimer diseases suffer from loneliness and isolation, which can easily affect their QoL and cognitive function [45],[46]. Future studies should assess the impact of different approaches used in a complex program on the various dimensions in adults with PD. The QoL results highlight certain limitations of group training, such as the ability to take the principle of training individualization into account. This is why some authors propose multidisciplinary interventions to better manage the symptoms of people with PD. For example, massage therapy could improve physical discomfort symptoms [47], whereas psychotherapy could help better manage anxiety and depression symptoms [48]. On the other hand, in a context of limited human and financial resources, the physical program proposed in the present study appears to meet several components necessary for the adequate care of persons with disabilities.

The low number of participants in the H&Y 3–4 group greatly limits the interpretation and comparison of groups according to the severity of PD. The exploratory findings indicated that both groups produced similar results, although those with a higher degree of severity (H&Y 3–4) achieved slightly greater improvements in mobility (TUG). The execution of complex tasks under supervision during all the intervention may have contributed to an increase in the sense of competence and confidence of the participants, which was reflected in a higher speed of execution of the TUG. However, the participants in the H&Y group 3–4 showed more severe symptoms; therefore, the possibility that they may have presented kinesiophobia at the beginning of the program, which could have reduced performance during the initial evaluation, should not be overlooked.

The data from this study did not confirm all the expected benefits for this type of activity. Although a community-specific physical program appears to have a stabilizing (maintaining) effect on physical capacity, in future research, it would be necessary to add a control group to facilitate the comparison and examination of the results. Furthermore, we believed that persons with mild to moderate conditions would react better than those with a higher severity score due to their cognitive reserve; it is clear that further research is needed in this area. It might be interesting to evaluate the long-term effect of a physical program with a higher training supply that would meet the recommendation of 3 to 5 times a week.

Furthermore, it is crucial to determine who prescribes physical exercises to older adults with PD and what solutions are available to slow down the progression of this disease. Neurologists are expected to provide the best physical programs and recommendations for patients with PD after identifying their specific needs. Nonetheless, physical educators and practitioners can also deliver diagnoses, initial treatments, and the regular and timely management of PD, most particularly in the clinical exercise field, where physical activity guidelines for populations with PD are still not very clear and need further research [49].

## 6. Conclusion

Epidemiological data has indicated that exercise may reduce the risk of developing PD. There is currently a solid body of clinical evidence demonstrating that PA is a helpful, cost-effective, and low-risk intervention that improves the general health and has the potential to alleviate both motor and cognitive symptoms in patients with PD [50]. A physical program appeared to be a good approach to improve and maintain the physical parameters and mental functions. While this study only displayed slightly significant benefits, further research on PA is required to determine the best forms of therapy in people with PD and across the spectrum of its symptom burden. This would be further supported by studies that indicate the positive association of PA and neural functions with the strong potential of this therapeutic modality to be better translated to and applied in the management of PD.
